# Association between 25-hydroxyvitamin D concentrations and pubertal timing: 6–14-year-old children and adolescents in the NHANES 2015–2016

**DOI:** 10.3389/fendo.2024.1394347

**Published:** 2024-05-22

**Authors:** Ziqin Liu

**Affiliations:** The Department of Endocrinology Children’s Hospital of Capital Institute of Pediatrics, Beijing, China

**Keywords:** 25(OH)D, pubertal timing, precocious puberty, national health and nutrition examination survey, NHANES

## Abstract

**Background:**

The association between 25(OH)D and pubertal timing has not been well studied. The aim of this study was to assess the relationship between 25(OH)D levels and pubertal timing in children.

**Methods:**

Participants aged 6–14 years who had available nutritional and serum sex hormone (total testosterone (TT) and estradiol (E2)) information (n =1318) were included. We conducted a cross-sectional analysis of the associations between 25(OH)D and sex steroid hormones among children in the National Health and Nutrition Examination Survey, 2015–2016. Puberty was indicated by high levels of steroid hormones (TT≥50 ng/dL in men, E2≥20 pg/ml in women) or menarche.

**Results:**

Serum 25(OH)D and pubertal status showed the same trend in both males and females. In the male population, the OR values of serum 25(OH)D between 50 and <75 and ≥75 nmol/L were 0.52 (0.25, 1.08) and 0.64 (0.23, 1.75), respectively, compared with serum 25(OH)D<50 nmol/L. The OR of serum 25(OH)D ≥50 nmol/L compared with <50 nmol/L was 0.54 (0.26, 1.10), and the P value was statistically significant (P=0.048). In the female population, when the serum 25(OH)D concentration was <50 nmol/L, the ORs corresponding to a serum 25(OH)D concentration between 50 and <75 and ≥75 nmol/L were 0.53 (0.29, 0.98) and 0.50 (0.19, 1.30), respectively. The OR of serum 25(OH)D≥50 nmol/L compared with <50 nmol/L was 0.52 (0.19, 0.96), and the P value was statistically significant (P=0.037).

**Conclusions:**

A lower 25(OH)D level was associated with earlier puberty in both girls and boys. There was a negative association between 25(OH)D concentrations and pubertal timing.

## 1 Introduction

Vitamin D and vitamin D receptor (VDR)-activating enzymes are expressed throughout the hypothalamus–pituitary–gonadal (HPG) axis (1, 2), and vitamin D is metabolized in the developing gonads (1, 3), suggesting a local role for vitamin D during development. The serum 25-hydroxyvitamin D (25(OH)D) concentration is used to evaluate individual vitamin D status and is the best indicator of vitamin D stores; indeed, it is the main circulating form of vitamin D and has a half-life of 2–3 weeks (4). The biological actions of vitamin D are mediated through the VDR, which is distributed across various tissues, including the skeleton and parathyroid glands, as well as reproductive tissues. The rich presence of the VDR in the hypothalamus is consistent with the distribution of other neurosteroids (5), and the VDR has been found in the human pituitary gland (6) as well as in the human endometrium (7). In women, VDR mRNA has been shown to be expressed in the ovaries (8). Peripubertal vitamin D3 sufficiency is important for an appropriately timed pubertal transition and maintenance of normal female reproductive physiology, and vitamin D3 is a key regulator of neuroendocrine and ovarian physiology (9). In men, VDR was detected in human testicular tissue homogenates using titrated vitamin D (10), and VDR was detected in human sperm, with binding sites in the nucleus and the midpiece of the sperm (11). The role of 25(OH)D in the activation of the HPG axis and in influencing the timing of puberty has been reported (12, 13). Furthermore, 25(OH)D has been found to be involved in the functioning of the reproductive system in several studies (1, 14, 15).

Puberty activates the HPG axis, leading to psychological and physical maturation, accelerated linear growth, the development of secondary sexual characteristics, and gonadal maturation (16). Common factors affecting pubertal development include genetics, environment, diet, and nutrition (17). Among the nutritional factors, vitamin D is very important. Vitamin D deficiency and insufficiency are global health issues that affect more than one billion children and adults worldwide (18). The role of vitamin D deficiency in puberty or precocious puberty remains controversial.

Considering that there are only a few relevant studies, which had small sample sizes and mostly included girls but not boys, we performed a literature search to provide evidence for the association between vitamin D and pubertal timing. To fill these knowledge gaps, we analyzed data on 6- to 14-year-old participants from the National Health and Nutrition Examination Survey (NHANES) and explored the effects of vitamin D status on puberty.

## 2 Methods

The datasets generated and analyzed in the present study are available at the NHANES website (https://www.cdc.gov/nchs/nhanes/index.htm). The NHANES is a nationally representative survey of the civilian, noninstitutionalized US population that was conducted by the National Center for Health Statistics (NCHS) of the Centers for Disease Control and Prevention (CDC). We downloaded data from one cycle of the NHANES from 2015 to 2016. The data contained five parts: demographic data, dietary data, examination data, laboratory data, and questionnaire data. All procedures and study procedures were approved by the NCHS Ethics Review Board, and written informed consent was obtained from all participants (19).

### 2.1 Study design and population

**Figure 1:**
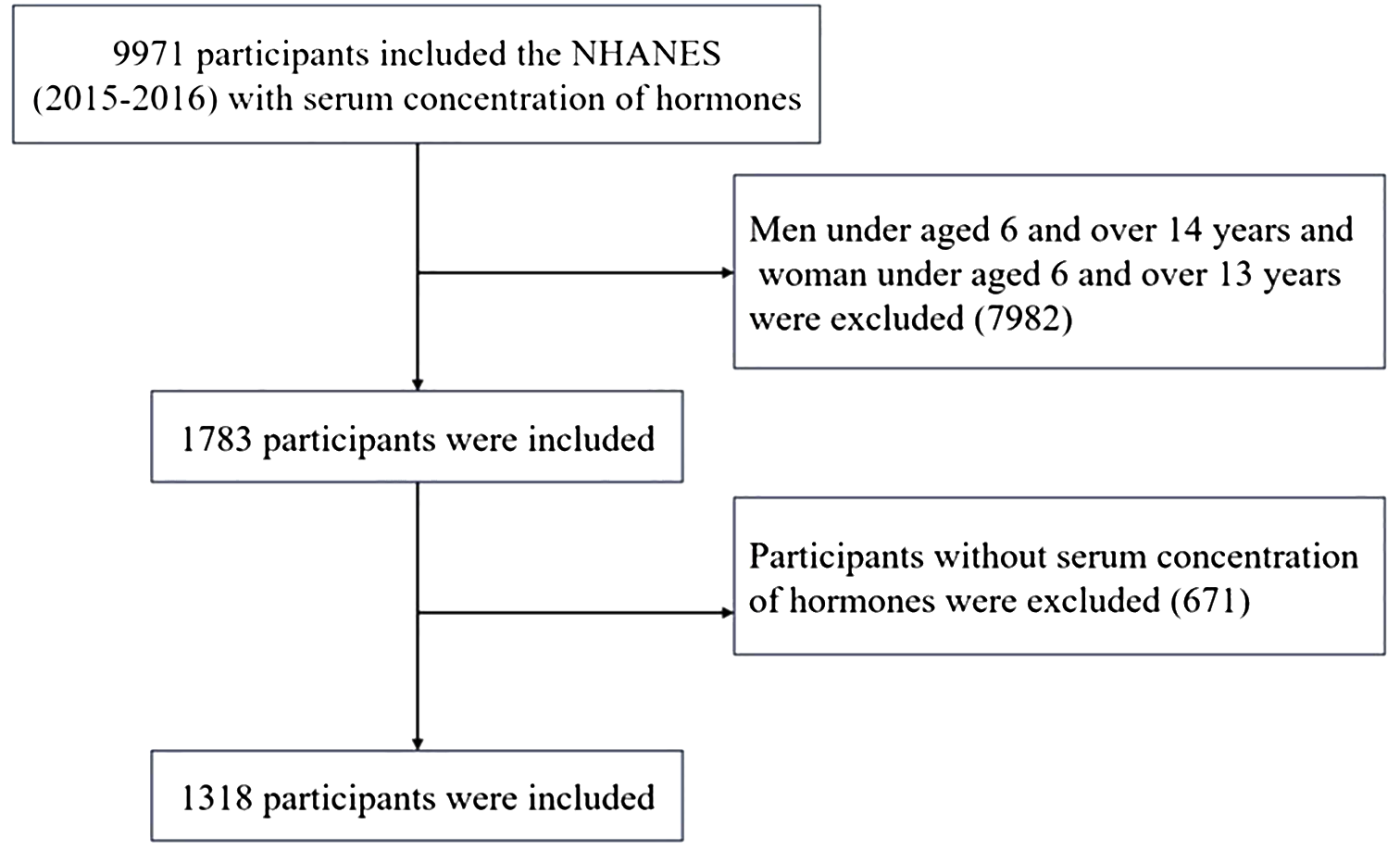
Flow chart of study participants.

A total of 1318 participants were included in this study. Participants who were younger than 6 years or older than 14 years of age (n = 7982) or who had missing data (n = 671) were excluded, while 1318 participants from the 2015–2016 NHANES who had available data on serum total testosterone (TT), estradiol (E2) and sex hormone binding globulin (SHBG) were included. The flowchart of the participant selection process is presented in **Figure 1**.

### 2.2 Measurement of sex hormone indicators

The test principle for the CDC method utilizes high-performance liquid chromatography−tandem mass spectrometry (HPLC−MS/MS) for the quantitative detection of 25(OH)D. Serum 25(OH)D levels were categorized according to the Endocrine Society Clinical Practice guidelines (17) as follows: <49.99 nmol/L was considered to indicate vitamin D deficiency; 50.00–74.99 nmol/L was considered to indicate vitamin D insufficiency; and ≥75.00 nmol/L was considered to indicate vitamin D sufficiency (20). Serum TT and E2 levels were measured using isotope dilution high-performance liquid chromatography tandem mass spectrometry (ID-LC−MS/MS), while the concentrations of SHBG were quantified based on the reaction of SHBG with antibodies and chemiluminescence measurements of the reaction products via a photomultiplier tube. The lower limits of detection (LLODs) for TT, E2 and SHBG were 0.75 ng/mL, 2.994 pg/m and 0.800 nmol/l, respectively. Puberty was indicated by high levels of steroid hormones (TT≥50 ng/dL in men, E2≥20 pg/ml in women) or menarche (21).

### 2.3 Statistical analysis

Descriptive statistics were used to analyze the characteristics and distribution of the population. Continuous variables are presented as the means ± standard deviations and were compared with t tests, whereas categorical variables are presented as counts (%s) and were compared with the χ2 test. The serum 25(OH)D concentration was included in the linear and multivariate logistic regression models as a continuous variable (increase in each SD) and as a categorical variable (tertiles, with the first tertile as the reference group). The adjusted variables for multivariate regression analysis were age, city of birth, serum high-density lipoprotein (HDL) level, serum total cholesterol (TC) level, and body mass index (BMI), BMI-SDS (obese: mean BMI SDS >+ 2SD, nonobese: mean BMI<=+ 2SD) and race. The downloaded data were visualized and analyzed using the statistical package R (R.4.3.1), with a two-tailed P<0.05 considered to indicate statistical significance.

## 3 Results

A total of 1318 participants (male: 711, female: 607) were included in this study, for which the male-to-female ratio was 1.17:1. The participant characteristics are shown in **Table 1**. The median levels of serum vitamin D were 65.0 ± 16.7 nmol/L and 62.8 ± 19.2 nmol/L in pubertal males and females and 58.3 ± 18.5 nmol/L and 53.8 ± 21.9 nmol/L in prepubertal males and females, respectively.

**Table 1:** Baseline demographic characteristics of the study population.

	Pubertal state(male)		*P*	Pubertal state(female)		*P*
	no	yes		no	yes	
number	485	226		391	216	
**age**	8.7±1.9	12.8±1.2	<0.001	8.2±1.7	11.5±1.2	<0.001
**Total family income($)**	10.6±11.9	10.6±12.5	0.983	11.9±16.3	10.6±13.7	0.338
**Country of birth**			0.012			0.022
Unite State	466(96.1)	206(91.2)		373(95.4)	195(90.3)	
Other country	19(3.9)	20(8.8)		18(4.6)	21(9.7)	
**Race**			0.360			0.251
Mexican American	104(21.4)	47(20.8)		102(26.1)	62(28.7)	
Other Hispanic	80(16.5)	30(13.3)		61(15.6)	25(11.6)	
Non-Hispanic White	131(27.0)	66(29.2)		102(26.1)	45(20.8)	
Non-Hispanic Black	98(20.2)	57(25.2)		77(19.7)	50(23.1)	
Other Race	72(14.8)	26(11.5)		49(12.5)	34(15.7)	
**Education (%)**			<0.001			<0.001
Less than 5^th^ grade	404(83.3)	16(7.1)		358(91.8)	55(25.5)	
5^th^ grade-9th	81(16.7)	210(92.9)		32(8.2)	161(74.5)	
Biochemical index						
HDL.mmol/L	1.5±0.4	1.4±0.3	<0.001	1.4±0.4	1.4±0.4	0.269
TC.mmol/L	4.1±0.7	3.8±0.7	<0.001	4.1±0.7	3.9±0.7	<0.001
LDL.mmol/L	1.9±0.5	2.1±0.7	0.360	2.8±0.5	2.2±0.8	0.032
Tg.mmol/L	0.5±0.2	0.8±0.7	0.176	1.2±0.4	0.8±0.5	0.068
VD.nmol/L	65.0±16.7	58.3±18.5	<0.001	62.8±19.2	53.8±21.9	<0.001
Physical examinations						
BMI.kg/m^2^	18.9±4.3	22.6±5.8	<0.001	18.9±4.4	22.1±4.9	<0.001
BMI SDS			<0.001			0.01
Obese (BMI SDS>2), n(%)	9(1.9)	26(11.5)		9(2.3)	15(6.9)	
Nonobese (BMI SDS≤2), n(%)	476(98.1)	200(88.5)		382(97.7)	201(93.1)	
Waist circumference.cm	65.9±12.7	78.7±15.5	<0.001	65.0±12.3	75.6±11.2	<0.001

Compared with prepubescent children, male children at puberty were much older; were from other countries; had a higher education level (grade 5 and above); had lower HDL (p<0.001), TC (p<0.001), and serum 25(OH)D levels (p<0.001); and had a greater BMI (p<0.001) and waist circumference (p<0.001). Female children at puberty were older than were those at prepuberty; were from other countries; had higher education levels (grade 5 and above); had lower levels of TC (<0.001), low-density lipoprotein (LDL) (<0.032), and serum 25(OH)D (<0.001); and had greater BMI (<0.001) and waist circumference (<0.001).

**Figure 2:**
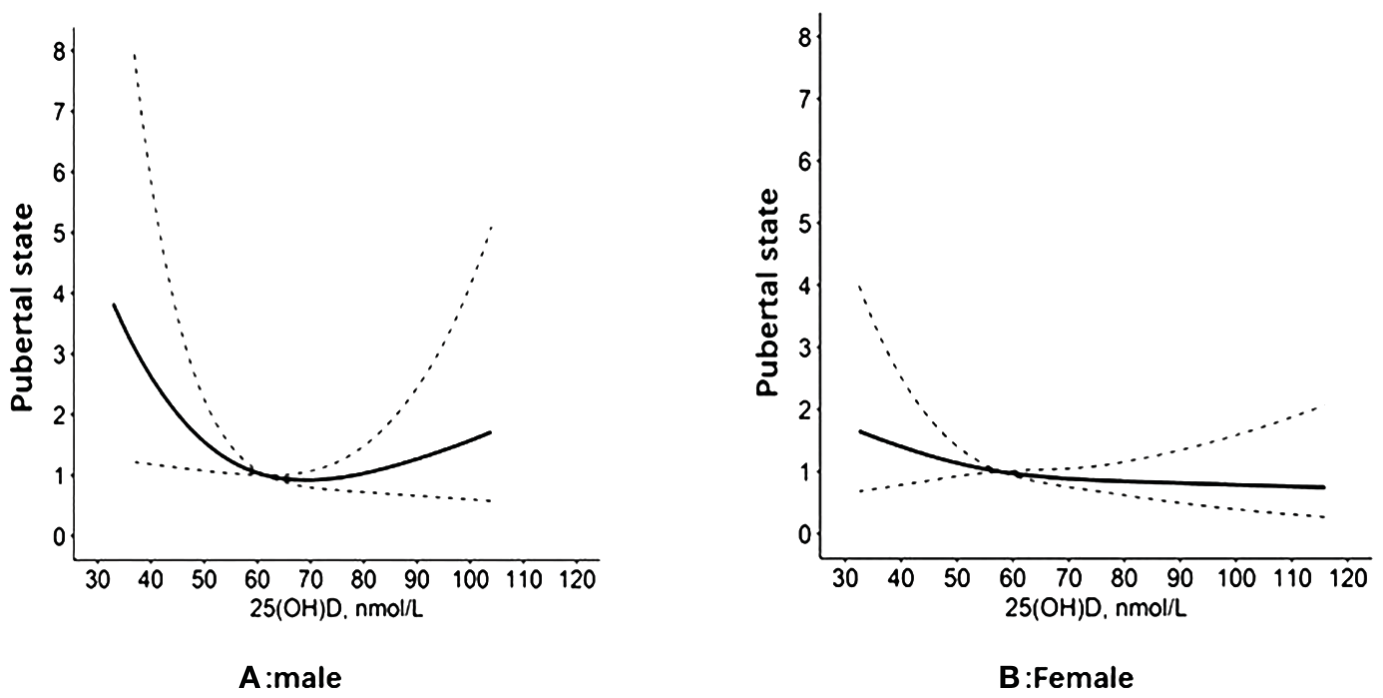
VD and the restricted cubic spline curve of pubertal state.

**Figure 3:**
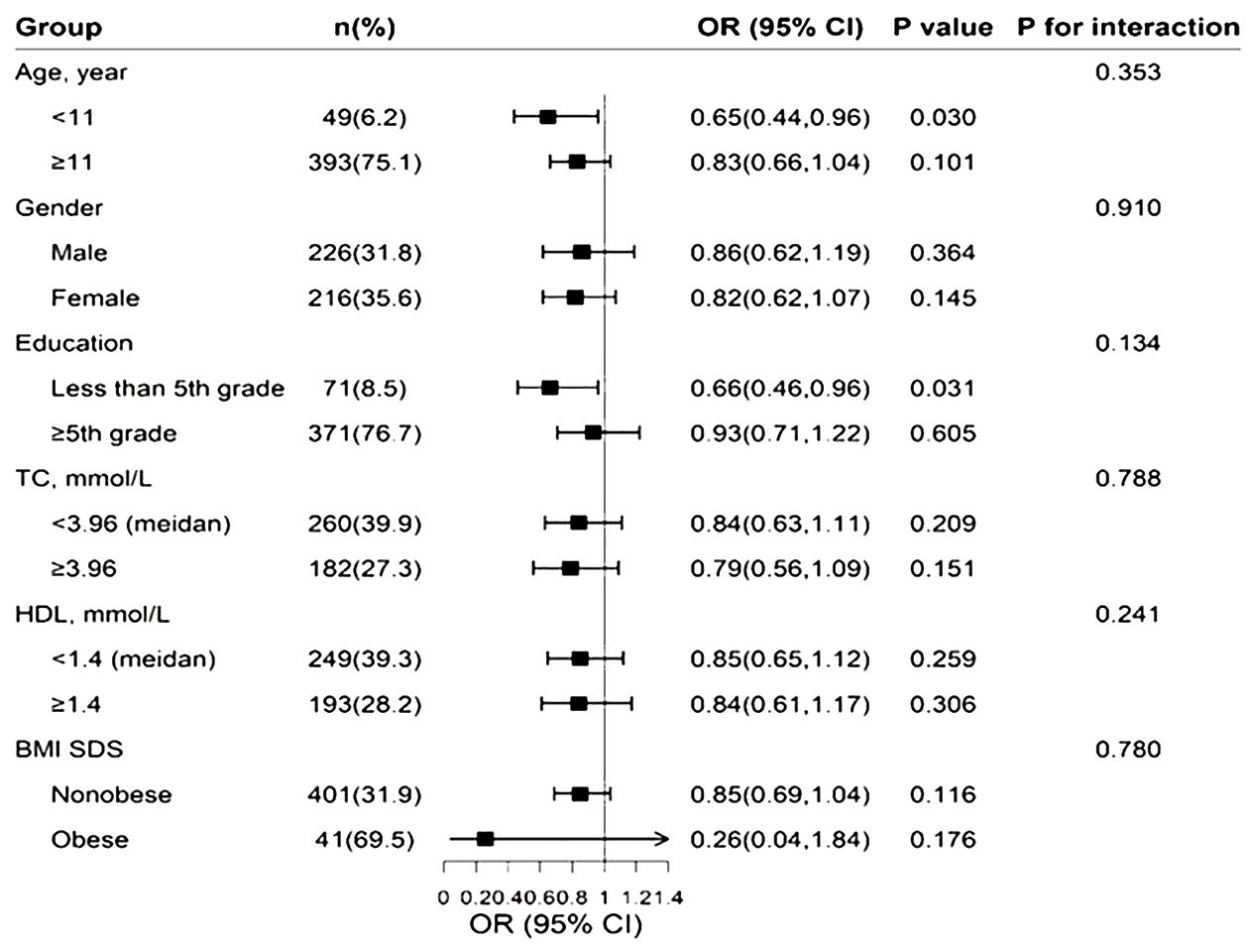
25(OH)D (each 1-SD increase) and pubertal state was analyzed by forest plots.

Regression analysis of serum 25(OH)D and pubertal status showed that after adjusting for relevant confounders (age, city of birth, education level, serum HDL levels, serum TC and BMI, race), there was no statistically significant association between one SD and the third quartile of serum 25(OH)D and pubertal status in either the male or female population. Serum 25(OH)D was further grouped according to clinical criteria <50, 50-<75 and ≥75 nmol/L. In the male population, the OR values of serum 25(OH)D and pubertal status between 50 and <75 and ≥75 nmol/L were 0.52 (0.25, 1.08) and 0.64 (0.23, 1.75), respectively, compared with serum 25(OH)D<50 nmol/L. The OR of serum 25(OH)D ≥50 nmol/L compared with <50 nmol/L was 0.54 (0.26, 1.10), and the P value was statistically significant (P=0.048). In the female population, when the serum 25(OH)D concentration was <50 nmol/L, the ORs corresponding to a serum 25(OH)D concentration between 50 and <75 and ≥75 nmol/L were 0.53 (0.29, 0.98) and 0.50 (0.19, 1.30), respectively. The OR of serum 25(OH)D≥50 nmol/L compared with <50 nmol/L was 0.52 (0.19, 0.96), and the P value was statistically significant (P=0.037). The results are shown in **Table 2**. The RCS revealed an inverse correlation between the serum 25(OH)D concentration and the odds of having reached puberty (**Figure 2**). Subgroup analysis of 25(OH)D was performed with forest plots of adolescent states, and no significant interaction factors were found (**Figure 3**).

**Table 2:** Logistic regression analysis of serum 25(OH)D and pubertal status.

VD (nmol/L)	N	Case (%)	Unadjusted model		Adjusted model	
			OR (95%CI)	*P*	OR (95%CI)	*P*
male						
Increase by 1SD	711	226(31.8)	0.66(0.55,0.79)	<0.001	0.86(0.62,1.19)	0.364
Tertiles						
<52.5	199	92(46.2)	ref		ref	
52.5-66.8	250	72(28.8)	0.47(0.32,0.70)	<0.001	0.56(0.27,1.16)	0.118
>66.8	262	62(23.7)	0.36(0.24,0.54)	<0.001	0.64(0.29,1.43)	0.280
Trend test				<0.001		0.274
Categories 1						
<50	156	74(47.4)	ref		ref	
50-<75	397	113(28.5)	0.44(0.30,0.65)	<0.001	0.52(0.25,1.08)	0.081
≥75	158	39(24.7)	0.36(0.22,0.59)	<0.001	0.64(0.23,1.75)	0.387
Trend test				<0.001		0.291
Categories 2						
<50	156	74(47.4)	ref		ref	
≥50	555	152(27.4)	0.42(0.29,0.60)	<0.001	0.54(0.26,1.10)	0.048
female						
Increase by 1SD	607	216(35.6)	0.63(0.53,0.76)	<0.001	0.82(0.62,1.07)	0.145
<52.5	239	119(49.8)	ref		ref	
52.5-66.8	188	59(31.4)	0.46(0.31,0.69)	<0.001	0.63(0.32,1.23)	0.177
>66.8	180	38(21.1)	0.27(0.17,0.42)	<0.001	0.53(0.25,1.16)	0.111
Trend test				<0.001		0.095
Categories 1						
<50	200	108(54.0)	ref		ref	
50-<75	298	83(27.9)	0.33(0.23,0.48)	<0.001	0.53(0.29,0.98)	0.042
≥75	109	25(22.9)	0.25(0.15,0.43)	<0.001	0.50(0.19,1.30)	0.154
Trend test				<0.001		0.068
Categories 2						
<50	200	108(54.0)	ref		ref	
≥50	407	108(26.5)	0.31(0.22,0.44)	<0.001	0.52(0.29,0.96)	0.037

Adjusted variables: age, city of birth, race, education level, serum high density lipoprotein, serum total cholesterol, BMI and BMI-SDS.

## 4 Discussion

In this study, the associations between 25(OH)D and pubertal timing were investigated, and a negative association was found between 25(OH)D and pubertal timing. This association was particularly strong among girls. In girls, 25(OH)D deficiency or insufficiency was associated with earlier puberty, and 25(OH)D deficiency was more likely to be associated with earlier puberty in boys. These findings could lead to clinical and dietary recommendations for individuals with 25(OH) deficiency and insufficiency in the childhood population. To our knowledge, this was the largest sample size in which the link between serum 25(OH)D levels and pubertal timing was examined in both males and females.

Puberty is the physiological process whereby adolescents reach sexual maturity and become capable of reproduction. Puberty onset varies naturally among individuals; in addition to genetic and environmental factors, the importance of nutritional factors, such as iron, zinc, calcium, and vitamin D, increases during puberty (4). 25(OH)D is a key regulator of neuroendocrine and ovarian physiology (5). However, the relationship between 25(OH)D status and pubertal timing is controversial. The findings of several studies are consistent with our results. For example, in one study, the probability of menarche was twice as high in vitamin D-deficient girls than in girls who were vitamin D sufficient. In girls, vitamin D deficiency has been shown to decrease the age of menarche by ∼10 months compared with that in girls with sufficient vitamin D (4, 22). A systematic meta-analysis of six studies showed that vitamin D-deficient individuals were more likely to develop precocious puberty (OR = 2.02 [95% confidence interval 1.65–2.46]) (4, 23). One retrospective study collected data from 221 girls with idiopathic central precocious puberty (ICPP) and 144 girls without ICPP, and the serum 25(OH)D levels in the ICPP group were significantly lower than those in the non-ICPP group (p < 0.001). A low serum 25(OH)D is an independent risk factor for ICPP (4, 24). Another meta-analysis showed that the average serum vitamin D concentration in individuals with precocious puberty was 1.16 ng/mL, which was lower than that in the control group (12).

However, in adult female mice, peripubertal 25(OH)D deficiency was associated with delayed puberty (5). In a study involving 713 (25.0%) Chinese men, participants with hypogonadism had significantly lower 25(OH)D levels and greater BMIs (25). In this study, both adolescent boys and adults were included. In another cross-sectional study, although females who had vitamin D deficiency were more likely to report an early age of menarche (i.e., at or before 9 years of age), this relationship disappeared after controlling for age at screening, race/ethnicity and BMI (4, 26). A retrospective study of 145 girls monitored for ICPP revealed that the mean 25(OH)D concentration was 27.6 ± 17.3 ng/mL, without any correlation with the pubertal characteristics of the subjects (4, 27). Another cross-sectional study showed no significant differences in 25OHD concentrations between ICPP patients and control participants. There were no significant differences in 25(OH)D concentrations between the CPP (25.4 ± 8.6 ng/mL) and control groups (28.2 ± 7.4 ng/mL) (28). However, most studies have described the association between vitamin D and menarche, which occurs in the middle or late stages of puberty. Therefore, one possible explanation for the differences between studies is that our study focused on the association between pubertal status and vitamin D levels in almost healthy participants.

The onset of puberty varies between males and females. Almost all previously published studies included only girls. However, whether vitamin D levels affect the occurrence of puberty and its role in males have been less studied. Our study revealed that the OR of serum 25(OH)D≥50 compared with <50 was 0.54 (0.26, 1.10), and the P value was statistically significant (P=0.048) in males, which was the same trend as that in females. Treatment with active vitamin D3 and VDR showed that VD3/VDR had a positive regulatory effect on Cyp11a1 expression and testosterone secretion. The VDR promotes testosterone synthesis in male mice by upregulating Cyp11a1 expression, which plays an important role in male reproduction (29). Testosterone is produced by Leydig cells and is responsible for male sex characteristics. LH induces steroidogenesis by increasing cyclic AMP production and the intracellular concentration of calcium ions (Ca2+) in Leydig cells, and 1,25-dihydroxyvitamin D3 might influence this calcium-dependent LH response (30, 31).

The prevalence of precocious puberty is sexually dimorphic and greater in girls than in boys (15–20 girls for every boy) (32). Based on our findings, it is possible that both males and females require vitamin D during puberty. Recently, several studies have shown that overweight and obesity are significantly associated with increased odds of ICPP among girls (33, 34). In a cross-sectional study of 220 females and 164 males (aged 7–16 years), vitamin D deficiency was found in 49% of the total patients and was significantly more prevalent in females than in males (33.1% in females; 15.9% in males, P < 0.001). Puberty is an additional risk factor for vitamin D deficiency, especially in girls and obese children (35). Our study showed that even after adjusting for BMI and BMI-SDS, vitamin D still plays an important role in pubertal timing, and that trend occurs in both sexes.

25(OH)D deficiency is prevalent worldwide, but the optimal concentration of serum 25(OH)D has not been determined. There are no data on how much vitamin D is required to prevent vitamin D deficiency in children aged 1–9 years, and no scientific evidence to date has demonstrated an increased requirement for vitamin D for children aged 9–18 years (24). Our study showed that vitamin D supplementation during peripuberty is equally important for males and females. The supplemental doses for males and females may need to be individualized.

There are several limitations to our study. First, since this was an observational study, cause and impact could not be determined. In addition, we did not perform a more detailed, stratified analysis by stage of puberty, and this subgrouping may have had a critical influence on the results.

## 5 Conclusion

After multivariate adjustment, there was a negative association between 25(OH)D concentrations and pubertal timing. These results highlight the potential advantages of monitoring and evaluating 25(OH)D status during puberty. The systemic effects of 25(OH)D have added another dimension to the endocrinology of puberty.

## Data availability statement

Publicly available datasets were analyzed in this study. This data can be found here: The datasets generated and analyzed in the present study are available at the NHANES website (https://www.cdc.gov/nchs/nhanes/index.htm).

## Ethics statement

All procedures and study contents were approved by the NCHS Ethics Review Board, and written informed consent was obtained from all participants. The studies were conducted in accordance with the local legislation and institutional requirements. Written informed consent for participation in this study was provided by the participants’ legal guardians/next of kin.

## Author contributions

ZL: Writing – original draft, Writing – review & editing.
